# Are different stoichiometries feasible for complexes between lymphotoxin-alpha and tumor necrosis factor receptor 1?

**DOI:** 10.1186/1472-6807-12-8

**Published:** 2012-05-08

**Authors:** Nahren Manuel Mascarenhas, Johannes Kästner

**Affiliations:** 1 Computational Biochemistry Group, Institute of Theoretical Chemistry, Pfaffenwaldring 55, University of Stuttgart, Stuttgart, 70569, Germany

**Keywords:** Elastic network model (ENM), Lymphotoxin, MM/PBSA, Receptor, Tumor necrosis factor (TNF)

## Abstract

**Background:**

Tumor necrosis factors, TNF and lymphotoxin-α (LT), are cytokines that bind to two receptors, TNFR1 and TNFR2 (TNF-receptor 1 and 2) to trigger their signaling cascades. The exact mechanism of ligand-induced receptor activation is still unclear. It is generally assumed that three receptors bind to the homotrimeric ligand to trigger a signaling event. Recent evidence, though, has raised doubts if the ligand:receptor stoichiometry should indeed be 3:3 for ligand-induced cellular response. We used molecular dynamics simulations, elastic network models, as well as MM/PBSA to analyze this question.

**Results:**

Applying MM/PBSA methodology to different stoichiometric complexes of human LT-(TNFR1)_n=1,2,3_ the free energy of binding in these complexes has been estimated by single-trajectory and separate-trajectory methods. Simulation studies rationalized the favorable binding energy in the LT-(TNFR1)_1_ complex, as evaluated from single-trajectory analysis to be an outcome of the interaction of cysteine-rich domain 4 (CRD4) and the ligand. Elastic network models (ENMs) help to associate the difference in the global fluctuation of the receptors in these complexes. Functionally relevant transformation associated with these complexes reveal the difference in the dynamics of the receptor when free and in complex with LT.

**Conclusions:**

MM/PBSA predicts complexes with a ligand-receptor molar ratio of 3:1 and 3:2 to be energetically favorable. The high affinity associated with LT-(TNFR1)_1_ is due to the interaction between the CRD4 domain with LT. The global dynamics ascertained from ENMs have highlighted the differential dynamics of the receptor in different states.

## Background

Protein-protein interactions are critical for signaling events within a cell. An investigation on the precise recognition of ligands by their respective receptors is an active field of research, since breakdown of such specific recognition is the root cause of several diseases and infections. One of the rational motives to understand such phenomena is to develop antibodies and small-molecule inhibitors that modulate the outcome of such interactions. One such system that generated immense attention owing to its central role in inflammatory effect, immunological response, but also in several autoimmune diseases and several pathogeneses is the tumor necrosis factor (TNF) [1,2]. Two TNF ligands, namely TNF-α (or TNF) and TNF-β (or lymphotoxin-α, LT) have been extensively studied to methodologically dissect cellular signaling and diseases related to their malfunction [3-6]. It is now well recognized that several cellular responses are directly dictated by TNFs and about 20 homologous cytokines have been identified [7].

**Figure 1:**
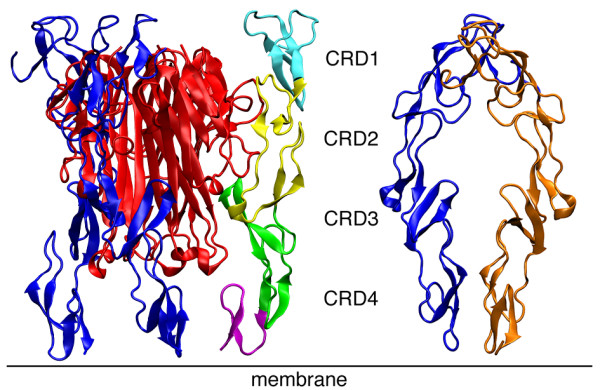
**Left: extracellular part of the complex of LT with three receptors, LT-(TNFR1)**_**3**_**; right: receptor dimer (TNFR1)**_**2**_**.** LT is colored red, the receptors blue. In the dimer complex, the receptor monomers are colored blue and orange. The locations of the CRDs of the receptors (color-marked in one receptor) are indicated.

TNF and LT exert their effects by binding to two receptors, TNFR1 and TNFR2 (tumor necrosis factor receptor 1 and 2) [8-10]. TNFRs are type I membrane receptors characterized by 2 to 6 CRDs (cysteine-rich domains) in the extracellular region of the receptor. Both TNFR1 as well as TNFR2 contain four CRDs. In solution as well as in their complex with receptors both TNFs exist as homotrimers and display similar secondary structure profile [11,12]. Their secondary structure is predominantly β-sheet with each monomer consisting of 8 anti-parallel β-strands. The β-sheets form a double layer, one facing the interior of the trimeric complex while the other is exposed to the solvent and is referred as “jellyroll” β-sheet sandwich. The outer β-sheet is hydrophilic while the interior sheets are mainly hydrophobic. TNFR1 on the other hand has an elongated structure with disulfide bridges between its domains. So far only the extracellular domain of the receptor, also called the soluble receptor, has been solved [13]. The X-ray structure of LT in complex with TNFR1 [12] proved vital in understanding how LT is recognized by its receptors. These bind at the grooves of the monomer-monomer interfaces of LT. Major contacting regions of TNFR1 lie at CRD2 and CRD3, see Figure 1. The recently solved X-ray structure of the TNF-TNFR2 complex [14] opens a new window of opportunities in this already interesting system. In the absence of ligands, receptors were found by crystallographic experiments as parallel or anti-parallel dimers [13], with the biological significance of the antiparallel dimerization mode being questionable. In the parallel dimer the ligand-binding domains are exposed to the solvent.

The TNFRs exhibit distinct functional roles and diverse signaling capabilities. While TNFR1 is expressed in all tissues, TNFR2 is expressed particularly in immune cells and other specialized cell types like endothelial and neuronal tissue [8,15]. TNFR1 primarily invokes cytotoxic activities of the cell whereas TNFR2 functions as a receptor for T-cell signaling and for mediating host infections [16]. In contrast to TNFR2, TNFR1 contains a death domain in its cytoplasmic region and upon ligand binding is capable of activating the apoptotic pathway [15]. Experimental evidence also suggests only TNFR2 to exhibit differential binding to soluble and membrane bound TNF-α [17]. Although the two receptors share good homology in their extracellular domain, their cytoplasmic regions show significant differences in their sequences. Due to their central role in cellular signaling, several diseases are directly linked to the TNF family of ligands and receptors [18]. Animal models of diseases have predicted the predominant role of TNFR1 in several pathogeneses and adverse causes of enhanced inflammation [19]. In contrast, TNFR2 has been demonstrated to be involved in defects related to cell-mediated immunological response [20]. Anti-TNF antibodies and engineered soluble TNFRs have been developed for the treatment of rheumatoid arthritis and other diseases [21-23].

Interaction of TNF with its receptors imparts a conformational change in the receptors that triggers a cellular response. But the precise mechanism of receptor activation by their ligand is still under debate. The first proposal for receptor activation, known as the ligand trimerization model, emphasizes the ligand to recruit the receptors to form the final complex with a ligand-receptor molar ratio of 3:3 [24]. Recent evidence has, however, raised serious questions on this mechanism of activation of the receptors. One school of thought envisages receptors as dimers or trimers in the absence of ligand and propose a pre-ligand assembly domain (PLAD) formed by the association of two or three receptors at the membrane distal CRD1 domain, prior to ligand binding [25,26]. Cross-linking experiments with TNFR1-Fas and TNFR2-Fas also suggest the formation of homodimers in the absence of a ligand [27]. These homo-dimers/trimers of receptors then build up to form cluster-aggregates on the cell surface upon ligand binding [28,29]. The CRD1 domain has also been shown to be important for stabilizing the CRD2 domain for efficient ligand binding [26]. The recent crystal structure of the CD40-CD154 complex in a 2:3 molar ratio has further hinted that the stoichiometry of TNF-TNFR complexes may not always be 3:3 [30]. Also, recent work has indicated that the formation of a trimer-monomer complex of a ligand trimer-receptor monomer complex of the TNF family member TRAIL is quite stable and may be the first step in the formation of the complex [31]. In this work we investigate the free energy of binding of LT-TNFR1 complexes. The objective of this work is to shed light on the way in which the receptors bind to the ligand and to estimate the free energy of binding involved in complex formation.

## Results

Molecular dynamics (MD) simulations have been carried out on the three stoichiometric complexes of LT with TNFR1, represented as LT-(TNFR1)_1/2/3_ along with their individual binding partners, monomeric TNFR1 (mTNFR1), the dimeric receptor ((TNFR1)_2_) and trimeric lymphotoxin (LT), each for 35 ns. The residues 28–171 of each chain of human LT and the residues 15–153 of human TNFR1 were included in our model. The receptor is made up of four cystein-rich domains: CRD1 (residues 15–53), CRD2 (54–97), CRD3 (98–138), and CRD4 (139–153). For analysis purposes and to have similar number of residues to those observed in the dimeric receptor (TNFR1)_2_ (PDB: 1NCF), only the residues 15–150 from TNFR1 were used in the MM/PBSA calculations and other analysis. Free energy of binding has been computed by using MM/PBSA methodology in single-trajectory and separate-trajectory methods. The components of free energies, gas-phase energies, and solvation free energies have been averaged over 1001 snapshots from MD trajectories.

**Figure 2:**
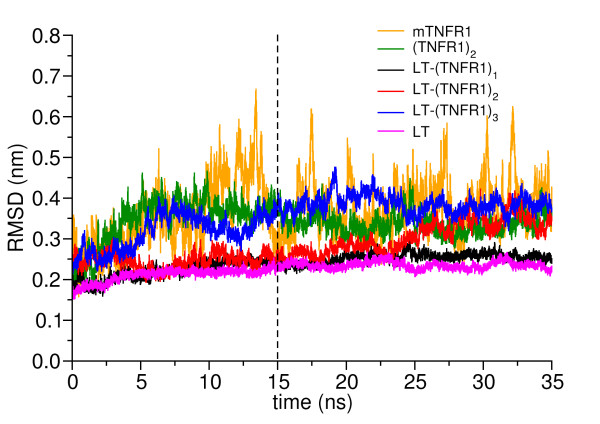
The root mean square deviation (RMSD) of the Cα atoms of the receptor as a function of time.

To ascertain the extent of deviation of the structure from their initial crystallographic conformation and to demonstrate proper equilibration, the time-dependence of the root-mean square deviation (RMSD) of Cα atoms was calculated with reference to the starting X-ray structure, see Figure 2. Since the terminal residues belonging to CRD3 and CRD4 domains, residues 134–153 of TNFR1, show high temperature factors and fluctuate readily in MD simulations, we omitted them in the RMSD calculations. All systems but mTNFR1 are well equilibrated within the simulation time scale. LT-(TNFR1)_1_ exhibits only minimal conformational transformation from its starting structure since its RMSD remains within 0.3 nm indicating it to be the most rigid among the studied protein-protein complexes. LT-(TNFR1)_2_ also follows a similar trend as exhibited by LT-(TNFR1)_1_. However, in the last 10 ns of the simulation there seems to be a slight increase in its RMSD value. On the other hand the LT-(TNFR1)_3_ complex exhibits a higher RMSD value and seems to equilibrate around 0.4 nm. The monomeric mTNFR1 displays a higher RMSD, which is expected since it is simulated in an unbound form and lacks any kind of external stabilization. The dimeric complex (TNFR1)_2_ also deviates from its initial structure by 0.35 nm, similar to LT-(TNFR1)_3_, and equilibrates after about 10 ns of simulation.

**Figure 3:**
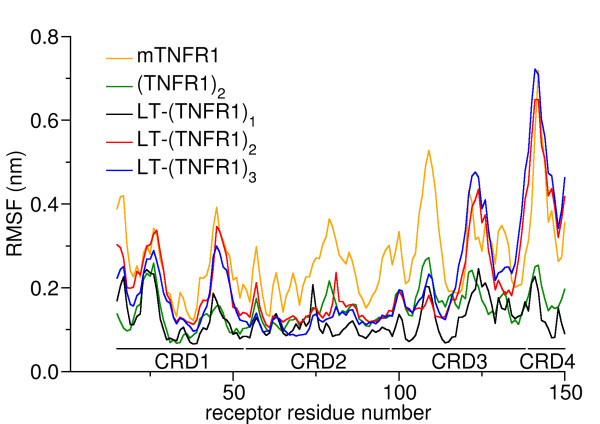
**The root mean square fluctuations (RMSF) of the Cα atoms of the receptor.** Regions with low RMSF indicate high stability.

To assess the dynamics associated with the receptor in ligand-unbound form, in dimeric form, and as complexes of different stoichiometries, the root-mean-square fluctuations (RMSF) of the Cα-atoms of the receptors were calculated, see Figure 3. The mTNFR1, as expected, exhibits relatively high peaks in the RMSF plots along its whole length, indicating its strongly fluctuating character. The dimer (TNFR1)_2_ is well stabilized by comparison, especially the residues in the CRD1 and CRD4 domains since these are the regions the two receptors interact with one another. Interestingly there seems to be immense stabilization even of the CRD2 and CRD3 domains in (TNFR1)_2_. Hence, the interaction in the CRD1 and CRD4 domains restrain the fluctuations of the CRD2 and CRD3 domains. These CRD2 and CRD3 domains of the receptor are the regions that are primarily involved in ligand binding in the LT-(TNFR1)_n_ complexes. Residues 77–81 and 107–114 of TNFR1 reside at the cleft formed by the interface between the LT monomers [12]. The fluctuations in these binding regions are, hence, well constrained in the dimer as well as in the complex compared to the monomeric form of the receptor. Further down we will discuss that Trp-107^TNFR1^ is one of the well-buried residues in the complex being sandwiched between the interface of the two monomeric ligands. The CRD4 had been claimed to be highly disordered [12,13] correspondingly huge fluctuations are observed for this region in our study. The later part of CRD3 and the whole CRD4 domain showed high levels of mobility in mTNFR1, LT-(TNFR1)_2_, LT-(TNFR1)_3_, but notably not in LT-(TNFR1)_1_. In fact in LT-(TNFR1)_1_ the residues in these regions display a similar profile to that observed for (TNFR1)_2_. Hence the immense stability attained by the CRD4 domains in LT-(TNFR1)_1_ indicates a different behavior compared to the LT-(TNFR1)_2_ and LT-(TNFR1)_3_ complexes which will be rationalized in more detail below.

Naismith and Sprang [32] have classified the structure of the receptor into two major types of sub-domains, namely A1 and B2 modules, based on the structural topology and on disulfide bridges. They denoted the receptor to be made up of three A1 and B2 sub-domains each, in the following order: A1 (residues 15–29), B2 (30–52), A1 (55–70), B2 (73–96), A1 (98–114), B2 (117–137), and A1 (139–153). These authors also relate the structure of TNFR1 to be similar to a spiral, where the B-modules correspond to the plates and the A-modules to the bolts about which they pivot. The dynamic cross-correlation matrix (DCCM) extracted for the receptors explicate the relations between these domains. The correlation patterns of mTNFR1, LT-(TNFR1)_2_, and LT-(TNFR1)_3_ are rather similar, but differ strongly from the pattern of LT-(TNFR1)_1_ (see Additional file 1: Figure S1). In the former, the B2 module of CRD2 and the A1 module of CRD3 (residues 73–96 and 98–114, respectively) are highly correlated. Both these modules are also anti-correlated to the B2 module (residues 30–52) of the CRD1 domain, to the CRD4 domain, and to some extent to the A1 module (residues 15–29) of CRD1. In contrast to LT-(TNFR1)_2_ and LT-(TNFR1)_3_, highly correlated fluctuations observed in mTNFR1 might be an artifact of its high flexibility since it is present in an unbound form. In mTNFR1, LT-(TNFR1)_2_, and LT-(TNFR1)_3_, the B2 module of CRD2 and the A1 module from CRD3 are highly correlated. Thus it can be argued that these sub-domains form a stable motif across these complexes. The loss of correlations in the dimer (TNFR1)_2_ is not surprising considering the interaction in this complex happens mainly via the CRD1 and CRD4 domains. However, the significant silencing of correlations in LT-(TNFR1)_1_ further supports a unique nature of the interaction between the LT and TNFR1 in LT-(TNFR1)_1_.

### Residues involved in binding

**Figure 4:**
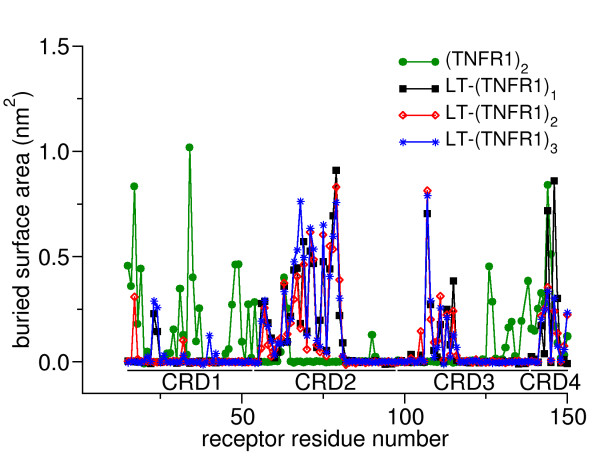
The buried surface area of the individual residues of the receptor in various complexes.

A qualitative measure of the underlying strength of interaction between two biomolecules can be gained by measuring the buried surface area of the complex. The buried-surface area of individual residues of the receptors in different complexes averaged over the trajectory is shown in Figure 4. In the LT-(TNFR1)_n_ complexes, residues in the CRD2 and CRD3 domains are buried while in the (TNFR1)_2_ complex residues in CRD1 and CRD4 are buried. It is not surprising that residues which are highly buried in the complexes with LT (in the domains CRD2 and CRD3) are highly exposed in (TNFR1)_2_. This gives valid proof in support of the argument that in the parallel form of the dimer the binding site domains are exposed to the solvent and can bind the approaching LT-ligands without any major structural change. In all LT-(TNFR1)_n_ complexes residues Leu71, Cys72, Arg77, Lys78, and Glu79 of CRD2 are strongly buried. In CRD3, residue Trp107^TNFR1^ is well buried and lies almost exactly at the interface between the chains of LT (at the membrane-proximal part of LT). In the LT-(TNFR1)_1_ complex two residues in CRD4, namely Phe144^TNFR1^ and Arg146^TNFR1^, are well buried indicating a strong interaction between the CRD4 domain and the ligand. In the (TNFR1)_2_ dimer, residues Gln17^TNFR1^, Lys19^TNFR1^, and His34^TNFR1^ in the CRD1 region and residue Phe144^TNFR1^ of the CRD4 domain are well buried.

### Hydrogen bonding

A good estimation of the polar interaction between two molecules can be made from estimating the hydrogen-bonding interaction between them. The hydrogen bonding interaction between the receptors in (TNFR1)_2_ and the receptor-ligand complexes in LT-(TNFR1)_n_ were measured purely based on the following geometric constraints using VMD [33]. A distance cutoff of 0.35 nm between the donor and acceptor with an angle cutoff of 60° in the angle donor-hydrogen-acceptor were defined to count for a successful hydrogen bonding interaction. The average number of hydrogen bonding interactions (per interface) over the trajectories was 25.5 for (TNFR1)_2_ and 31.4, 25.7, and 27.7 for LT-(TNFR1)_1_, LT-(TNFR1)_2_, and LT-(TNFR1)_3_, respectively. Hence, the interaction between the receptor and the ligand is strongest in the LT-(TNFR1)_1_ complex while it is pretty similar within all other complexes.

### Complex structures and receptor motions

**Figure 5:**
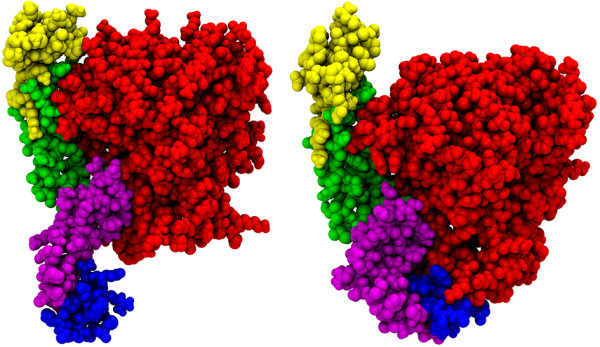
**Starting structure (left) and final simulated structure (right) of LT-(TNFR1)**_**1**_**.** LT is shown in red, the receptor CRDs 1 to 4 are colored yellow, green, purple, and blue. The figure demonstrates the attachment of the CRD4 domain to the LT.

The LT-(TNFR1)_3_ complex crystallizes as a trimer and hence one would expect it to be the most stable complex with minimal fluctuating character. On the contrary, the analysis performed so far highlights the immense stability of LT-(TNFR1)_1_ among the LT-(TNFR1)_n_. This is interesting considering one recent investigation of the stability of various stoichiometric complexes of TRAIL-(DR5)_n_ concluded the TRAIL-(DR5)_1_ complex to be more stable than the corresponding dimeric and trimeric complexes [31]. Figure 5 shows the starting and the final structures from MD of LT-(TNFR1)_1_. It was found that the CRD4 domain of TNFR1 in LT-(TNFR1)_1_ bends towards the homotrimeric LT to form a stable interaction with its residues.

**Figure 6:**
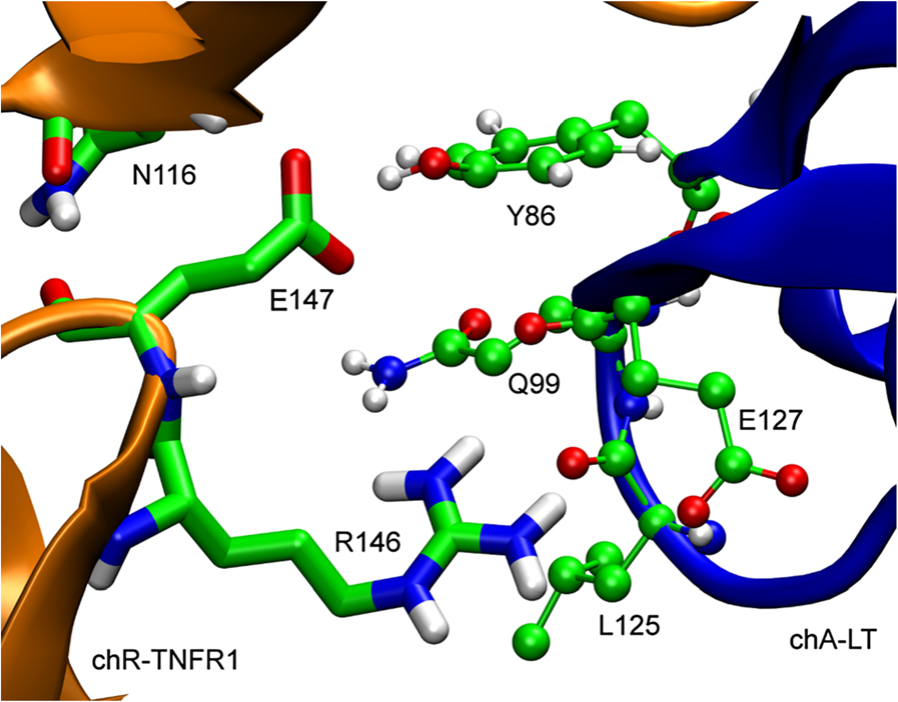
**The major interaction sites of CRD4 with LT in the complex LT-(TNFR1)**_**1**_**.** Residues of TNFR1 (left, backbone colored orange) are drawn as sticks, while those of LT (right, backbone colored blue) are represented as ball-stick models.

**Figure 7:**
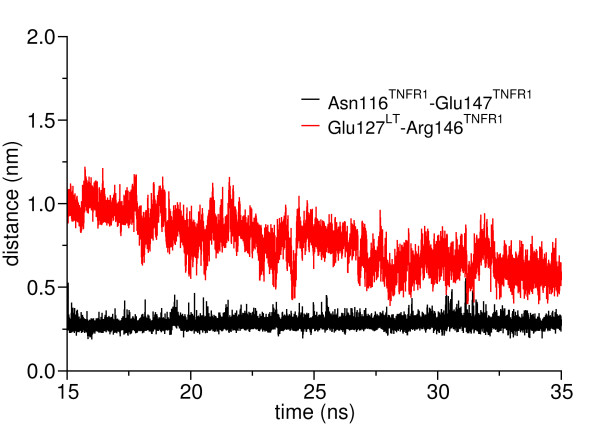
**Distance between the peptide-H of Asn116**^**TNFR1**^**and Cδ of Glu147**^**TNFR1**^**as well as Cδ of Glu127**^**LT**^**and Cζ of Arg146**^**TNFR1**^**during the simulation.** These hydrogen bonds stabilize the CRD4 at the membrane-proximal part of LT.

In order to investigate the residues that contribute to the interaction between CRD3 and CRD4 of TNFR1 and LT in the LT-(TNFR1)_1_ complex, the hydrogen bonding interactions between these were investigated. In about 94% of the snapshots a hydrogen bond between the side chain of Tyr86^LT^ and that of Glu147^TNFR1^ was observed. Residue Arg146^TNFR1^ was also observed to interact with several residues of LT through hydrogen bonding interactions: Leu125^LT-chA^, Gln126^LT-chA^, Glu127^LT-chA^, and Tyr122^LT-chB^ where chA corresponds to one chain of LT, chB to the neighboring chain, see Figure 6. Considering that Arg146^TNFR1^ and Glu147^TNFR1^ are stronger buried in LT-(TNFR1)_1_ than in LT-(TNFR1)_2_ and LT-(TNFR1)_3_ it is not surprising that these are involved in strong hydrogen bonding with the ligand. These two hydrogen bonding interactions remain fairly strong throughout the simulation and contribute significantly to the binding of CRD4 of TNFR1 to LT in the LT-(TNFR1)_1_ complex. The bending of the CRD4 domain is further stabilized by a strong internal hydrogen bond between Glu147^TNFR1^ and the backbone of Asn116^TNFR1^. Initially the distance between the two atoms Glu147-Cδ^TNFR1^ and the peptide-H of Asn116^TNFR1^ is 1.4 nm. It decreases to about 0.25 nm during the last 20 ns of the simulation indicating the strong interaction between the two residues. Arg146^TNFR1^ and Glu127^LT-chA^ form a salt bridge. The distance between the two side-chain atoms Cδ and Cζ, of Arg146^TNFR1^ and Glu127^LT-chA^ are plotted as function of time in Figure 7. It is obvious that the distances remain within the range of a strong salt-bridge.

**Figure 8:**
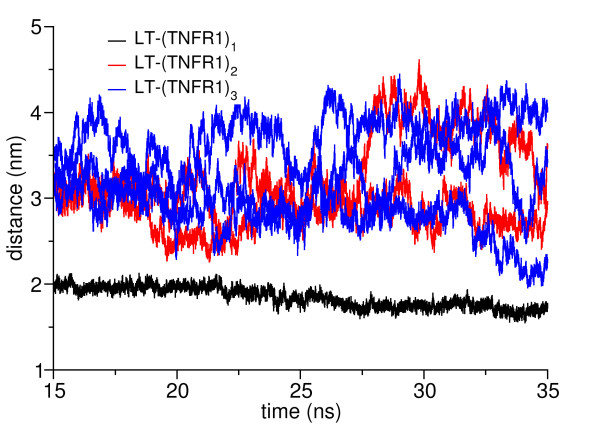
**The distance between the CRD4 domain of the receptor (TNFR1) and the center of LT during the simulation in the three different complex stoichiometries.** It can clearly be seen that CRD4 attaches to LT in LT-(TNFR1)_1_ while it remains unbound in LT-(TNFR1)_2_ as well as LT-(TNFR1)_3_.

The CRD4 domain has been observed to fluctuate readily in these complexes. Hence, to ascertain the motion of this domain, the distance between the center of LT and the center of the CRD4 domains were monitored for the whole 20 ns of one set of production runs (15–35 ns) (Figure 8). The distances between them fluctuates strongly in all complexes except LT-(TNFR1)_1_. In LT-(TNFR1)_2_ as well as LT-(TNFR1)_3_ the domains move independently from each other: while one CRD4 of LT-(TNFR1)_2_ remains at about 2.8 nm distance from LT, the other moves significantly further away, to about 3.5 nm. The same holds true for LT-(TNFR1)_3_. In LT-(TNFR1)_1_, however, the CRD4 is immobilized close to LT, at distances between 2.0 and 1.7 nm. To confirm this result, we ran additional simulations. Within 35 ns each, the CRD4 domains did not attach to LT in LT-(TNFR1)_2_ and LT-(TNFR1)_3_. In two of the three additional simulations carried out for LT-(TNFR1)_1_, the CRD4 domain attached to LT after 14 ns and 24 ns, respectively, confirming our proposal of the attachment of CRD4 to LT. Only in one simulation did it not attach within this simulation time. We assume that longer simulations would clarify this issue. This explains the distinct role of LT-(TNFR1)_1_ compared to the other complexes already found by the alternative analysis tools. The inability of the CRD4 domain to bind to LT in LT-(TNFR1)_2_ and LT-(TNFR1)_3_ can be explained on the basis of their structure. The three LT monomers are arranged in a triangular cone with their narrow ends pointing at the membrane. The width of LT at the top site is about 50 Å while it is only 30 Å at the membrane proximal region [12]. Hence the space at the bottom of the LT is quite small (Figure 1). The space on the LT is probably only adequate to accommodate one CRD4 domain. In LT-(TNFR1)_2_ and LT-(TNFR1)_3_ it may be that the competition between the CRD4 domains prevents either of them to bind.

### Results from the elastic network model

One of the major drawbacks of MD simulations is that a system needs to be simulated for long time scales to arrive at a meaningful interpretation of functionally relevant motions. This naturally requires computational time ranging from weeks to months for systems like the one studied here. In order to overcome such time-consuming calculations several coarse-grained computational methods have been developed. One such model that has received wide popularity is the elastic network model (ENM). Several studies have shown the low-frequency normal modes obtained from ENM to capture the conformational transition of several biomolecules which have been summed up nicely in the following reviews [34,35]. Hence, ENM is considered a powerful tool to establish the large-scale motions of proteins. One factor that dictates the outcome of the ENM is the spring constant for the interacting atoms. Several groups have explored distinct ways to rationalize their choice of force constants [36,37]. In this work, as discussed in Methods, we defined a set of three force constants depending on the nature of the bonds. It is to be noted that the ENM was constructed based on the X-ray structures and is, thus, independent of the results of the MD simulations.

**Figure 9:**
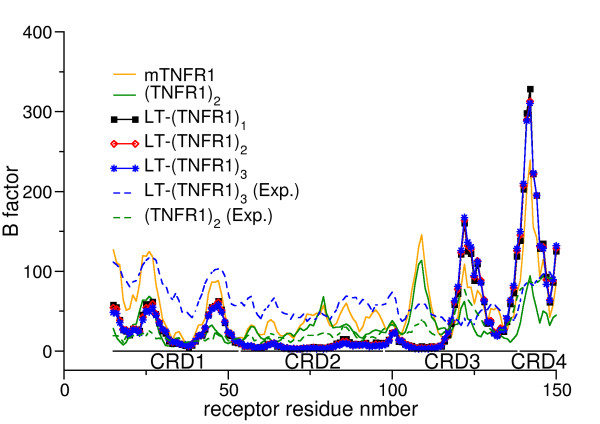
**Comparison of the B factors (temperature factors) as obtained from crystallographic structures (1TNR**[[12]]**and 1NCF**[[13]]**, respectively) to those obtained from an ENM.**

The low-frequency normal modes obtained from ENMs have been shown to be functionally relevant motions of the protein in many cases [38]. As a first method of validation of the results obtained from the ENM, one usually compares the fluctuating nature of the individual residues as ascertained from the ENM to that obtained from the experimental temperature factors (Figure 9). Here, we additionally compare it to the fluctuations found in MD. The rapidly fluctuating segments of the protein as obtained from the MD simulation agree well with those obtained from the ENM. The fluctuations appearing at the CRD1 domains are reduced for (TNFR1)_2_ compared to LT-(TNFR1)_3_. This is expected since CRD1 is the main interaction site in (TNFR1)_2_. The CRD4 domains on the other hand fluctuate in a similar fashion for the (TNFR1)_2_ and LT-(TNFR1)_3_ complexes in experiment (temperature factors in the crystal structures). But results from ENM paint a completely different picture. The CRD4 domains are the most fluctuating domains of the receptors, corroborating with our results from MD simulations (Figure 3). One major difference between MD and ENM, though, is that the CRD4 domain of the receptor in LT-(TNFR1)_1_, which is the least fluctuating LT-(TNFR1)_n_ complex in the MD simulations, also displays huge fluctuating behavior in ENM. This can clearly be rationalized by the fact that CRD4 is immobilized and bound to LT in LT-(TNFR1)_1_ in the MD simulations while it is unbound in the ENM at the crystal structure.

**Figure 10:**
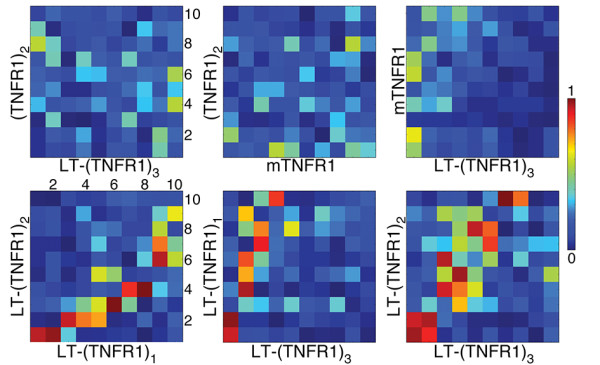
**Overlap between the first 10 eigenmodes of the receptor in different complexed or monomeric states.** These data are obtained from an ENM. Strong overlap indicates similar receptor dynamics.

Having compared the fluctuations of the receptors in different states, we now compare the conformational changes of the receptor in different states as estimated from the ENM normal modes. This was accomplished by comparing the overlap of the first 10 vibrational eigenmodes (Figure 10). The term “overlap” here refers to the dot product of the two eigenvectors. A high degree of overlap of the eigenmodes indicates that both proteins explore a similar conformational space. The RMSD between residues 15–150 of the receptors in LT-(TNFR1)_3_ (PDB: 1TNR) and (TNFR1)_2_ (PDB ID: 1NCF) is just 1.65 Å, indicating little geometrical difference between these states. Hence one is tempted to speculate that the receptors in the two forms would exhibit similar conformational changes. In contradiction, comparison of the eigenmodes shows that the global fluctuations of the receptors are well distinguished in the two forms (Figure 10). Also between the two ligand-unbound forms of the receptor, namely, mTNFR1 and (TNFR1)_2_, no significant overlap of the eigenmodes was found. This shows that the global fluctuations of the receptors do not resemble each other in the monomeric and the dimeric forms. The corresponding overlap between LT-(TNFR1)_3_ and mTNFR1 is also weak, indicating the ligand-binding to cause completely different receptor-motions than in both ligand-unbound forms. Comparison of the overlap between LT-(TNFR1)_3_ and LT-(TNFR1)_2,1_ indicates how ligand-binding transforms the receptor to a diverse range of conformational transformations. The binding of just one receptor to the ligand improves the overlap observed for the eigenmodes of the receptors to LT-(TNFR1)_3_. The binding of the second receptor improves this correlation even more. A very high degree of overlap is observed for the first two eigenmodes of LT-(TNFR1)_2_ and LT-(TNFR1)_3_. The third eigenmode from LT-(TNFR1)_3_ overlaps with the the forth eigenmode of LT-(TNFR1)_2_ and vice versa (Figure 10). Since the lowest-frequency normal modes are the most significant functional motions of the protein, the high degree of overlap between LT-(TNFR1)_2_ and LT-(TNFR1)_3_ shows that the dominant motions of the receptors are similar in these two complexes. These results give an overall perspective of the difference in receptor’s functional motions upon ligand binding.

**Figure 11:**
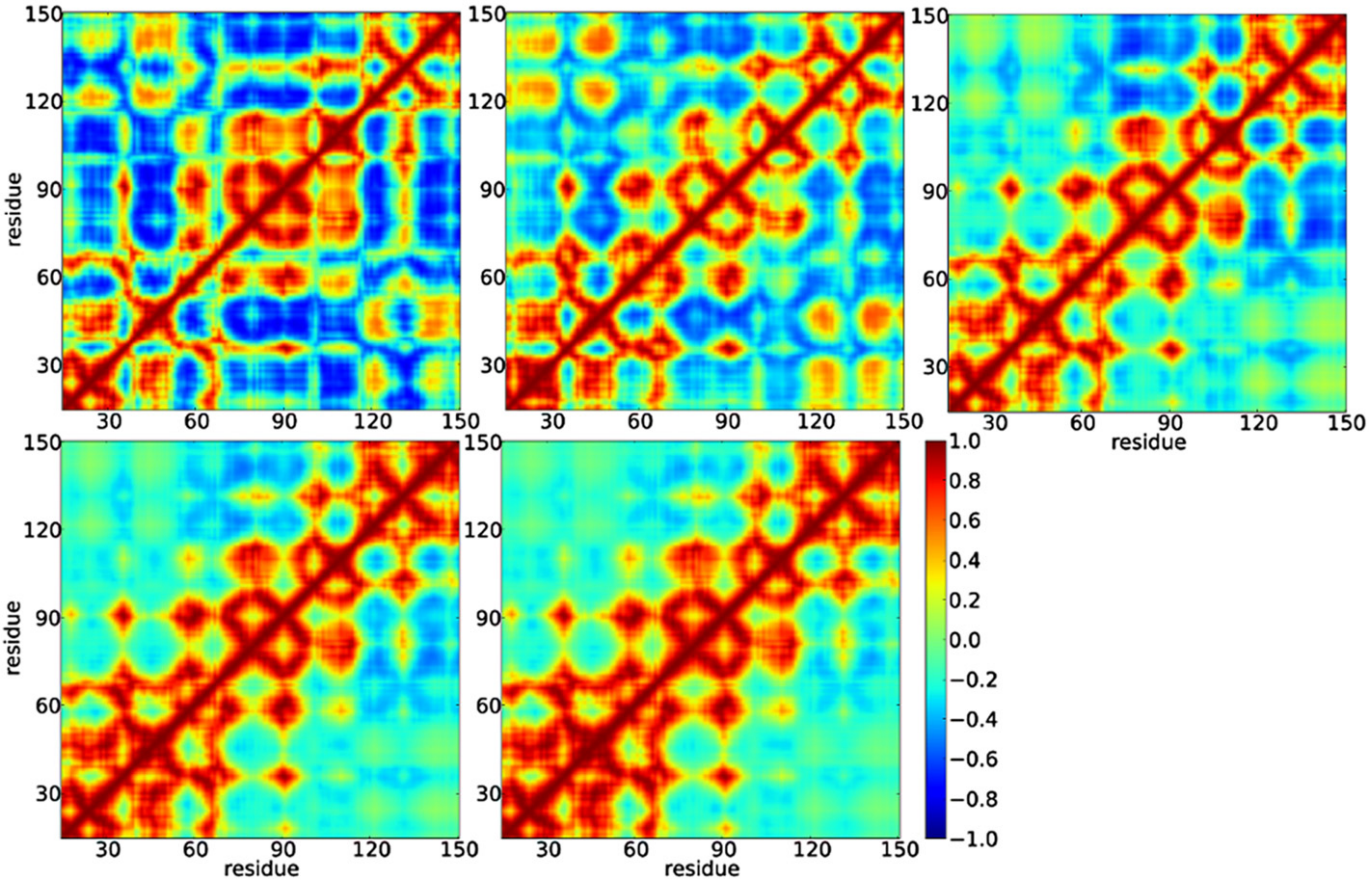
**The correlation matrix calculated from the 25 lowest-frequency normal modes of the ENM.** Top row: mTNFR1, (TNFR1)_2_ , LT-(TNFR1)_1_. Bottom row: LT-(TNFR1)_2_, LT-(TNFR1)_3_.

A correlation analysis clearly shows the difference between the two ligand-free forms of the receptors (Figure 11). The anti-correlated regions observed for mTNFR1 are somewhat diminished for (TNFR1)_2_. For example the anti-correlations observed between residues 15–34, 37–54 and 75–114 are significantly reduced. Also the anti-correlated motions between the residues 15–34 and 124–138, visible for mTNFR1, are completely lost in (TNFR1)_2_. Another interesting aspect to be extracted from this comparison is that the strong correlations observed within residues 74–114 in mTNFR1 is lost in (TNFR1)_2_. However, the correlations within residues 54–114 in (TNFR1)_2_ resemble those of the LT-(TNFR1)_n_ complexes. Thus, the dynamic motions of the CRD2 and CRD3 domains, which form the ligand-binding domains of TNFR1, fluctuate in a similar fashion in (TNFR1)_2_ and LT-(TNFR1)_n_. This suggests that the CRD2 and CRD3 domains in (TNFR1)_2_ are optimally aligned and fluctuate in manner that the ligands can easily identify them. From these conclusions it can be speculated that ligands prefer to bind to the receptor in its dimeric form rather than to monomers. The receptors in their ligand-bound forms resemble each other very closely except that the anti-correlations of the CRD2,3 domains with CRD3,4 are somewhat diminished in LT-(TNFR1)_2_ and more so in LT-(TNFR1)_3_. Also in ligand-bound forms of the receptor, a strong correlation seems to prevail within the CRD1 domain as well as within the second-half of CRD3 and CRD4 domains. Such correlations are more strongly observed for LT-(TNFR1)_2,3_ than for LT-(TNFR1)_1_.

**Figure 12:**
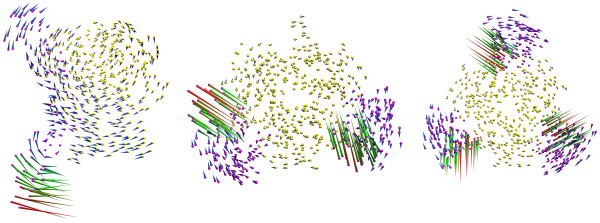
**The dominant functional motions as extracted from the lowest frequency normal modes obtained from the ENM.** Only the Cα atoms of the receptors (purple) and LT (yellow) are shown. The arrows indicate the dominant motion. They are colored in the increasing magnitude of displacement from their initial structure in the order blue-green-red. Left: LT-(TNFR1)_1_ side view, membrane at the bottom; middle: LT-(TNFR1)_2_ top view; right: LT-(TNFR1)_3_ top view.

The most dominant functional motion as extracted from the lowest-frequency normal mode of the elastic network model (ENM) is shown as arrows in Figure 12. The lengths of the arrows are proportional to the magnitude of the fluctuation of the residues. The vectors are scaled to result in an RMSD of 2 Å between the elongations in both directions. In all complexes the strongest fluctuations are exhibited by residues of the CRD4 domain. The ligand hardly contributes to this motion. The first mode extracted from LT-(TNFR1)_1_ is predominantly a hinge-bending motion. The overall motion drives the CRD4 domain towards the membrane-proximal center of LT. Notably, the CRD4 domain exhibits a similar bending motion in the MD. Hence, the results from the ENM are in good agreement with the results obtained from MD. The dominant motions of LT-(TNFR1)_2_ and LT-(TNFR1)_3_ are also concentrated at the CRD4 domains, but their direction is perpendicular to the direction of motion observed for CRD4 in the LT-(TNFR1)_1_ complex.

### Free energy of binding

In this work the formation of LT-(TNFR1)_3_ was split into the following three fundamental steps in accordance with the trimerization model.

Step 1.

(1) LT +12TNFR12→→ LT-TNFR11

Step 2.

(2) LT−TNFR11+12TNFR12→→ LT-TNFR12

Step 3.

(3) LT−TNFR12+12TNFR12→→ LT-TNFR13

Our motive for applying the MM/PBSA method on this system was to shed light on the stability of LT-(TNFR1)_n_ complexes of different stoichometry. Though precise estimations of binding free energies for protein-protein complexes are tough, results from MM/PBSA are known to correlate well with experimental binding free energies [39]. The precise mechanism for the activation of TNFR1 has been subject to immense debate. The previous belief of a 3:3 molar ratio of the ligand-receptor has been hugely influenced by the first crystallographic structure of the LT-(TNFR1)_3_ complex. In the recent past, however, evidence and arguments have been presented that question if indeed that should be the case. Recently Reis et al. [31] showed for the TRAIL-DR5 system, a system similar to LT-TNFR1, that the affinity of DR5 for TRAIL is strongest for the binding of the first receptor molecule compared to the binding of second and third, suggesting a ligand-receptor molar ratio of 3:1. Another family of TNF-receptor systems, the CD154-CD40, crystallizes in the molar ratio 3:2 [30]. Hence, it is worth to analyze if such 3:1 and 3:2 stoichiometric complexes are stable and plausible for LT-(TNFR1)_n_. The major advantage of the MM/PBSA method is its ability to determine free energies with relatively low computational expense coupled with the advantage of breaking down the free energy components into different energy terms obtained from molecular mechanics and solvation. Nevertheless, the MM/PBSA analysis presented here should more be understood as providing qualitative insight rather than quantitative numbers.

### Results from single-trajectory simulations (SITA)

For the calculation of free energy components of the binding energy from MD simulations, one needs to extract the coordinates of the individual binding partners as well as the complex. It is possible to obtain the coordinates of the individual proteins from a single simulation of the complex, which is referred as the single-trajectory approach (SITA). Alternatively, when individual MD runs have been performed on the individual binding partners and their complex separately, we refer to them as separate-trajectory approach (SETA). One major advantage associated with SITA is the reduction in the computational requirement since only a single simulation of the complex needs to be performed. But this approach is valid only if the binding partners do not undergo major conformational and dynamic changes upon complex formation. In the present system, the receptors exhibit huge fluctuations and domain movements as discussed above.

The long-range electrostatic interactions hugely influence protein-protein complexes. Hence accurate estimation of these influences is eminent in any free energy method. One usually estimates the accuracy of these calculations based on the trade-off between the gas-phase electrostatic interaction (∆H_elect_) and the polar contribution to solvation (∆G_polar_) obtained from Poissan-Boltzmann (PB) calculations. The total electrostatic interaction (∆H_elect_ + ∆G_polar_) is a compromise between the electrostatic energy between the individual protein in the complex and the cost associated with desolvation of the respective proteins. Hence a positive value indicates the cost of desolvation is higher than the electrostatic interaction between the binding partners to form the complex. In all the complexes considered in the present study, it seems that electrostatic interaction disfavors protein-protein binding. For example, from the SITA an electrostatic interaction of −668.7 kJ/mol in (TNFR1)_2_ is lost due to a higher polar solvation energy of 709.3 kJ/mol, resulting in an unfavorable total electrostatic interaction of 40.6 kJ/mol, see Table 1. In the same manner an unfavorable total electrostatic interaction (∆H_elect_ + ∆G_polar_) of 24.4 kJ/mol, 22.4 kJ/mol, and 17.6 kJ/mol is found for LT-(TNFR1)_1_, LT-(TNFR1)_2_, and LT-(TNFR1)_3_, respectively. Hence, from SITA one can conclude that the polar contributions to the free energy only disfavors interaction between the two binding partners. The apolar contribution to solvation free energy (∆G_apolar_) on the other hand is favorable across these complexes. The van der Waals interaction between the proteins (∆H_vdW_) is very high in all these complexes indicating that such non-polar interactions contribute majorly to complex stability. Therefore it is safe to conclude that the total non-polar components contribute favorably towards binding free energies while the overall electrostatic term disfavors complex formation.

**Table 1:** Binding energies (in kJ/mol) obtained from single-trajectory analysis

	**(TNFR1)_2_**	**LT-(TNFR1)_1_**	**LT-(TNFR1)_2_**	**LT-(TNFR1)_3_**
∆H_vdW_	−671.6 ± 1.2	−739.5 ± 2.2	−600.7 ± 1.7	−670.4 ± 1.9
∆H_elect_	−668.7 ± 3.5	−676.5 ± 4.3	−567.4 ± 4.6	−662.8 ± 4.2
∆G_polar_	709.3 ± 3.3	700.9 ± 4.0	589.9 ± 2.0	680.4 ± 1.8
∆G_apolar_	−60.7 ± 0.1	−73.4 ± 0.2	−59.3 ± 0.1	−66.7 ± 0.2
∆G_binding_	−691.6 ± 5.0	−788.5 ± 6.3	−637.5 ± 6.4	−719.4 ± 6.2

The actual numbers of the binding energies are certainly too large (too negative). However, their relative magnitude is more reliable and allows to draw qualitative conclusions.

### Results from separate-trajectory simulations (SETA)

We have additionally used the SETA method to calculate the total free energy of binding in these complexes. There are two ways one can extract the energy components of TNFR1, either from TNFR1 or from (TNFR1)_2_. (TNFR1)_2_ was chosen since crystal structures suggest receptors to exist as dimers in the absence of ligand. Table 2 shows the components of the free energy obtained from separate-trajectory simulations. In all complex formations except that of LT-(TNFR1)_2_ the internal energy obtained from the force field is negative, i.e., binding of two receptors to LT is favorable while binding of one and three receptors to LT is not. This can be explained by the unfavorable conformational strain caused by the bending of the CRD4 of TNFR1 towards LT in LT-(TNFR1)_1_. In LT-(TNFR1)_2_, the binding of the second receptor relaxes the strain since both CRD4 domains of TNFR1 are unbound again.

**Table 2:** Binding energies (in kJ/mol) obtained from SETA

	**Step 1**	**Step 2**	**Step 3**
∆H_int_	94.4 ± 4.1	−44.3 ± 4.6	110.0 ± 4.9
∆H_vdW_	−202.2 ± 5.0	−21.4 ± 6.3	−169.7 ± 6.9
∆H_elect_	−434.2 ± 12.5	−183.0 ± 13.4	−257.6 ± 13.5
∆G_polar_	461.6 ± 11.0	143.3 ± 11.7	267.0 ± 11.8
∆G_apolar_	−36.4 ± 0.3	−13.6 ± 0.3	−36.0 ± 0.2
∆G_solv_	425.2 ± 11.0	129.7 ± 11.7	231.1 ± 11.8
∆G_gas+solv_	−116.8 ± 17.9	−119.0 ± 19.4	−86.2 ± 19.8

The actual numbers of the binding energies are certainly too large (too negative). However, their relative magnitude is more reliable and allows to draw qualitative conclusions.

The free energy of binding, ∆G_gas+solv_, estimated from SETA for LT-(TNFR1)_1_, LT-(TNFR1)_2_ and LT-(TNFR1)_3_, is −116.8, −119.0 and −86.2 kJ/mol, respectively. The electrostatic interaction between receptor and ligands is quite high for these complexes. However, the total electrostatic interaction (∆H_elect_ + ∆G_polar_), which is the sum of the contribution of electrostatic interaction between the binding partners and the solvation energy, gives a true picture of the electrostatic interaction between the proteins in the complex. The values for the steps 1 to 3 in this investigation are 27.3, −39.7, and 9.3 kJ/mol. Hence, binding of the second receptor to LT is electrostatically favorable in contrast to binding of first and third receptors. The non-polar interaction between the receptor and the ligand is negative; for the binding of second receptor the value is comparatively less pronounced. All this suggests that the binding of second receptor imparts a significant change to LT-(TNFR1)_1_.

A range of forces and constraints are at play when two proteins interact to form a complex. The conformational freedom of the individual binding partners varies between the complex and their free form. A parameter that reflects conformational restrain is the change in the internal energy. When this parameter is positive it indicates the binding partners have to be conformationally constrained to form the complex while a negative value indicates that the conformational restrains on the individual binding partners have been relaxed. For steps 1 and 3 the ∆H_int_ values are positive while for step 2 it is negative, indicating that binding of two receptors to the ligand is favored. The association of two capable binding partners occurs invariably at the cost of entropy. Entropic changes are hard to estimate in MM/PBSA. However, in our case in each of the three steps the receptor from free solution binds to the ligand. The major contribution to entropy arises then from the loss in entropy of the receptor from its state free in solution to the state bound to the ligand. Since we mainly compare the free energies of the different stoichiometric complexes, the entropy contribution arising from this step should then be comparable and cancels in the differences. For this reason we have ignored entropic contributions in the free energy calculations. The free energy of binding (ΔG_gas+solv_) values obtained from our study suggest a stoichiometric ratio 3:1 and 3:2 are of similar stability and are little higher in comparison to a 3:3 complex, suggesting such complexes are energetically feasible.

## Discussion

In this work, we tried to judge using MM/PBSA methodology if LT-(TNFR1)_n=1,2,3_ complexes can form with a ligand-receptor molar ratio of 3:1 and 3:2. The exact mechanism of receptor activation is still unknown. In accordance with the ligand trimerization model, the free energy of binding involved in the sequential binding of the receptors has been estimated. Using an ENM the global fluctuations that are associated with these complexes have been investigated. The results from MD simulations of the three stoichiometric complexes of the receptor with LT reveal that the CRD4 domain is attached to LT and stabilized in LT-(TNFR1)_1_ while it exhibits extensive fluctuations in the other two complexes. The low-frequency normal modes as observed from ENM analysis display highly symmetric motions of the three CRD4 domains in the LT-(TNFR1)_3_ complex. A direct impact of these motions on the cytoplasmic domains can be postulated. It has been recently proposed that six Fas intracellular death domains come in close proximity for inducing the formation of the oligomeric complex of Fas molecules [40]. Motions as observed in our ENM might be necessary and appropriate for aligning the intracellular domains in a systematic fashion and with right steric requirements to activate the signaling cascade. Such domain motions in tandem can change between an activated state, where they bring the intracellular domains to correct proximity, and a moderately active or inactivate state, where such proximity between the intracellular domains is either partially or completely lost.

In accordance with TRAIL-DR5[31], we observed that LT-(TNFR1)_1_ complex is stable, which is proposed to arise from the binding of the CRD4 domain to LT. We observed that only in the LT-(TNFR1)_1_ complex, the CRD4 domain binds to LT. Three residues, namely Phe144, Arg146, and Glu147 have been determined to be crucial for such an interaction. If such an interaction leads to a stable LT-(TNFR1)_1_ complex, as predicted from our MM/PBSA studies, it opens the debate if the LT-(TNFR1)_1_ complex represents an inactive state of the receptor. While LT-(TNFR1)_1_ would be inactivate the binding of subsequent receptors could lead to its activation. Our results from ENM indicate quite similar domain motions of the receptors in LT-(TNFR1)_2_ and LT-(TNFR1)_3_ which differ from those in LT-(TNFR1)_1_.

The results of the free energy of binding, ΔG_gas+solv_, estimated from MM/PBSA from single-trajectory analysis reveal the LT-(TNFR1)_1_ complex to be the most stable among LT-(TNFR1)_n=1,2,3_ while that from separate-trajectory analysis suggest LT-(TNFR1)_n=1/2_ to be equally stable. Although both methods utilize the same complex trajectory as input, only in SETA does one include the coordinates of ligand and protein in their unbound form from independent simulations. Since the receptors undergo a huge conformational change upon complex formation, the results from SETA should be more trustworthy. Several factors have a direct effect on the results of MM/PBSA, which include the force-field used, simulation time, charge models, solute dielectric constant and the surface boundary [41-43]. While the actual numbers obtained may be too high, their relative magnitude is expected to be more reliable. Estimation of free energies of binding for a protein-protein complex is a tedious task. Two major bottlenecks need to be overcome in such simulations, sufficient sampling and accurate estimation of entropy. The energy values obtained from this study are from 20 ns of data which we believe are a good compromise between size of the system and the number of simulations that needs to be undertaken coupled with the corresponding computational cost. The objective of this investigation was to get a hint whether ligand binding in a sequential fashion, as in steps 1, 2, and 3 leading to the final 3:3 complex strengthens or weakens the protein-protein interaction. The absolute numbers might not be that relevant but their relative values aid in better understanding of the interaction of the protein-protein complexes in different stoichiometry. In that sense our free energy results suggests both LT-(TNFR1)_1_ and LT-(TNFR1)_2_ to be more stable than LT-(TNFR1)_3_.

## Conclusion

The present study provides new insight into the LT-(TNFR1)_n_ complexes. The CRD4 domain of the receptor in the LT-(TNFR1)_1_ complex was observed to bind to LT. With the aid of ENM models the functional motions exhibited by LT-TNFR1 complexes have been portrayed. Our analysis suggests the CRD4 to exhibit a kind of zig-zag motion in LT-(TNFR1)_2_ and LT-(TNFR1)_3_ but to be well immobilized in LT-(TNFR1)_1_. The low-frequency normal modes derived from ENM analysis also support the CRD4 domain to be involved in highly fluctuating motions. Our free energy results based on MM/PBSA calculations on single-trajectory and separate-trajectory support the proposal of stable LT-(TNFR1)_1_ and LT-(TNFR1)_2_ complexes.

## Methods

### System setup

We model the interactions of LT with TNFR1. The starting structure for our simulation was the crystallographic structure of the human LT-(TNFR1)_3_ complex (PDB ID: 1TNR) [12] and the receptor dimer (TNFR1)_2_ (PDB ID: 1NCF) [13]. Other structures for the simulations, LT, TNFR1, LT-(TNFR1)_1_, and LT-(TNFR1)_2_ were extracted from the trimeric complex structure of LT-(TNFR1)_3_. All simulations were performed with GROMACS (ver. 4.0.7) [44] using the Gromos 43a2 (united-atom) force-field [45]. The proteins were placed such that a minimum distance of 0.7 nm is ensured between any sides of the dodecahedral unit cell and protein atoms. Proteins were then solvated in water modeled as simple point charge (SPC) [46]. To preserve electro-neutrality Na^+^ or Cl^–^ ions where added when necessary. The whole setup was energy minimized, first with steepest decent followed by conjugate gradient. As the first step in molecular dynamics, random velocities were generated at 300 K. Keeping the heavy atoms of the protein restrained with a force constant of 1000 kJ mol^-1^ nm^-2^, the solvent molecules were allowed to equilibrate for 30 ps. The restraints were then removed and system was further allowed to equilibrate at 300 K for another 1 ns. The simulation was then extended to 35 ns of MD run. The temperature was maintained at 300 K using the Berendsen thermostat [47] with a coupling constant of 0.1 ps. Protein and solvent were independently coupled to the reference temperature. In the first equilibration phase a Berendsen barostat was used while in the subsequent MD run Parrinello–Rahman [48] pressure coupling (with a coupling constant of 1 ps) was applied. Short-range non-bonded interactions were cut off at 1.2 nm. Electrostatic interactions above this range were evaluated using PME. The pair list was updated every 5 steps. All bonds were constrained using the LINCS algorithm [49] permitting an integration time step of 2 fs.

### Estimation of the free energy of binding by MM/PBSA

MM/PBSA (Molecular Mechanics/Poisson-Boltzmann Surface Area) is one of the simplest and yet most widely accepted free-energy estimation method. MM/PBSA combines molecular mechanics energies and continuum solvent approaches to predict free energies of binding [50]. The method has found widespread use since its introduction more than a decade ago. It has also been applied to protein-protein complexes [39]. According to this approach, the free energy of binding (ΔG_binding_) may be evaluated as

(4) $$\Delta {\text{G}}_{\text{binding}}=\Delta {\text{G}}_{\text{complex}}–\Delta {\text{G}}_{\text{protein}}–\Delta {\text{G}}_{\text{ligand}}$$

where each individual term on the right hand side of equation (1) is made up of the following terms,

(5) $$\text{G }={\text{H}}_{\text{gas}}+{\text{G}}_{\text{solv}}–\text{TS}$$

(6) $${\text{G}}_{\text{solv}}={\text{G}}_{\text{polar}}+{\text{G}}_{\text{apolar}}$$

(7) $${\text{H}}_{\text{gas}}={\text{H}}_{\text{int}}+{\text{H}}_{\text{vdW}}+{\text{E}}_{\text{elect}}$$

(8) $${\text{H}}_{\text{int}}={\text{H}}_{\text{bond}}+{\text{H}}_{\text{angle}}+{\text{H}}_{\text{dihedral}}$$

In the above expressions H_bond_, H_angle_, and H_dihedral_ are the contributions to internal energy (H_int_) obtained from the components of potential energy of the force field. The energy terms H_vdW_ and H_elect_ are van der Waals and electrostatic interaction energy, respectively. H_elect_ was computed using the coulomb module of the APBS software [51]. The H_vdW_ energies were computed fully, i.e., without either periodic boundary conditions or cutoff using GROMACS. The free energy contribution from solvation (G_solv_) is estimated from the polar (G_polar_) and apolar (G_apolar_) contributions to the solvation. The energy terms are averaged over 1001 equally spaced snapshots extracted from the last 20 ns of the molecular dynamics trajectory. VMD version 1.9 was used for visualization and for the hydrogen bond analysis [33].

The electrostatic component of the solvation free energy G_polar_, resulting from the Poisson-Boltzmann equation, was calculated with the program APBS. In this study the PARSE parameters were used [52]. The interior relative dielectric constants of the protein and the solvent dielectric were set to 2 and 78.54, respectively. The van der Waals surface was used for the dielectric boundary. 225 grid points in each direction and a grid spacing of 0.5 Å were used for all calculations. No counterions were included for the calculation. The non-polar contributions to solvation were estimated from the solvent accessible surface area (SASA), ${\text{G}}_{\text{apolar}}=\gamma \text{ SASA}+\text{b}$, where γ = 0.0227 kJ mol^-1^Å^-2^, b = 3.85112 kJ mol^-1^[52]. The SASA of the solute molecules were calculated using APBS. The objective of the study was to compare the free energies of the different stoichiometric complex. Since estimating entropy contribution to binding in a protein-protein complex is a challenging task, especially for a protein of this size we have ignored entropy contributions to free energy.

### Elastic Network Model (ENM)

Coarse-grained elastic network models (ENM) have gained enormous attention in the past decade to study the intrinsic motions of a protein [34]. Two commonly used ENM models are the Gaussian network model (GNM) [53] and the anisotropic network model (ANM) [54]. In this work we used an ANM to extract the functionally relevant motions exhibited by the protein. In a conventional ANM analysis, only the Cα atoms are considered and connections between them are defined based on a cutoff. In this study we have incorporated few extensions to this general approach. (a) In addition to the Cα atoms the side chains of the residues were also included in this model at a coarse-grained level. Hence, for every residue (except for glycine) two nodes have been defined, one at the Cα and the other at the center of mass of the heavy atoms of the side chain. A similar strategy has been previously adopted on the chaperonin GroEL [55]. For the residues ASP, ASN, ARG, LYS, GLN, and GLU, which have their interaction center primarily at the terminus of the side chain, we used the Cγ, Cγ, Cζ, Nζ, Cδ, and Cδ positions, respectively, instead of their side chain center. (b) Several types of force constants have been assigned between atoms depending on their bonding criteria or distance. A side chain node is attached to its Cα with a force constant of 10 kcal mol^-1^ Å^-2^. For atoms that are within 0.4 nm, between 0.4 and 0.8 nm, and between 0.8 and 1.2 nm, a force constant of 3, 2, and 1 kcal mol^-1^ Å^-2^, respectively, was assigned. Interactions between atoms further than 1.2 nm apart were ignored. (c) The receptors in this study possess several disulfide bonds, which act as major forces that render stability to the receptor. Hence, including these interactions in ENMs was considered imperative. To identify these disulfide bridges we used the DSSP program [56]. A force constant of 10 kcal mol^-1^ Å^-2^ was assigned between the side chain nodes of the two cysteine residues to mimic the disulfide bridge. (d) Secondary structural information was included by raising the force constant between Cα atoms in the backbone of α-helices and β-sheets to 6 kcal mol^-1^ Å^-2^. Structural elements were identified using the program STRIDE [57]. We calculated the Hessian matrix with the 'pdbmat' program [58] and the matrix was diagonalized using Prody [59] to calculate the eigenvalues and eigenvectors and to perform further analysis related to ENM.

## Competing interests

The authors declare that they have no competing interests.

## Authors’ contributions

Both authors planned and organized the project. NMM performed the calculations and the analysis, and drafted the paper. Both authors revised the paper, read and approved the final manuscript.

## Supplementary Material

Additional file 1: Supporting Information [60]. Click here for file
