# Supplementary material for: Are different stoichiometries feasible for complexes between lymphotoxin-alpha and tumor necrosis factor receptor 1?
Source: BMC Struct Biol. 2012 May 8;12:8. doi: 10.1186/1472-6807-12-8 (PMC3412742; doi:10.1186/1472-6807-12-8)
Supplement: Additional file 1: — Supporting Information [60]. [file 1472-6807-12-8-S1.pdf]

Supporting Information to

Are different stoichiometries feasible for complexes between lymphotoxin-alpha and tumor necrosis factor receptor 1?

Nahren Manuel Mascarenhas and Johannes Kästner

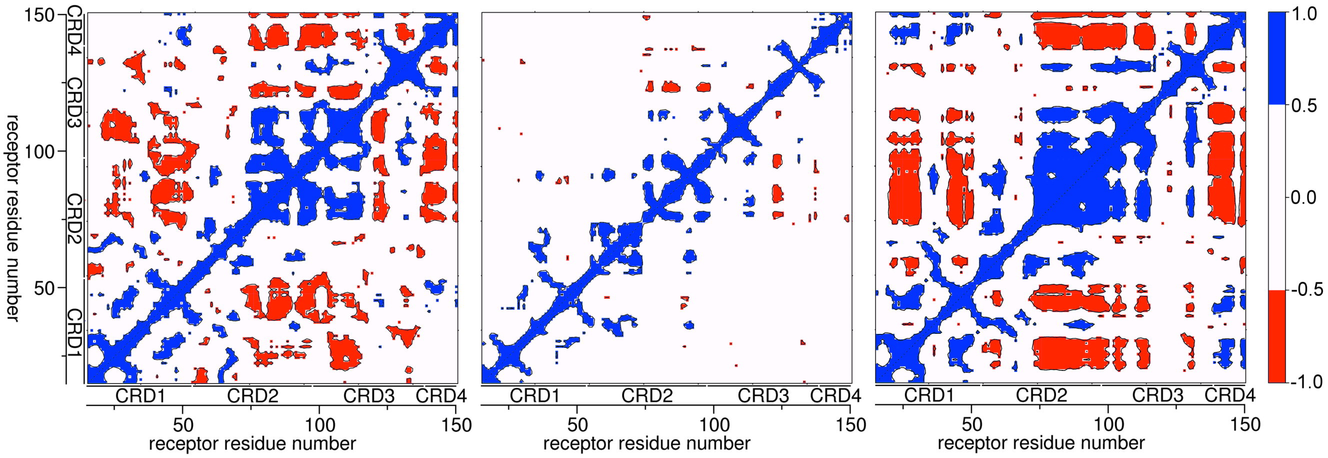

Fig. S1: The dynamic-cross correlation matrix (DCCM) plots [S1] of mTNFR1, LT-$(TNFR1)_1$ and LT-$(TNFR1)_3$.

S1. Grant BJ, Rodrigues AP, Elsawy KM, McCammon JA, Caves LS: **Bio3d: an R package for the comparative analysis of protein structures** *Bioinformatics* 2006, **22**:2695-2696.
